# No U‐turn on sodium reduction

**DOI:** 10.1111/jch.14021

**Published:** 2020-08-29

**Authors:** Laura K. Cobb, Thomas R. Frieden, Lawrence J. Appel

**Affiliations:** ^1^ Resolve to Save Lives, an initiative of Vital Strategies New York NY USA; ^2^ Johns Hopkins University Baltimore MD USA

## 1 INTRODUCTION

High blood pressure is the world's leading modifiable risk factor for preventable mortality, causing more than 10 million deaths per year,^1^ more than the number of deaths from all infectious diseases combined. Sodium reduction has the potential to prevent millions of cardiovascular events and deaths, primarily through reducing blood pressure,^2^ and tested, scalable approaches exist to reduce sodium in commercially processed packaged foods. However, implementation has been limited by two problems: (a) academic controversy, with a few vocal authors mistakenly suggesting that low levels of sodium can increase cardiovascular mortality,^3^, ^4^ and (b) the challenges of implementing population‐wide sodium reduction interventions.^5^ This article suggests ways forward on both issues.

## 2 ACADEMIC CONTROVERSY

Although there is broad consensus that sodium reduction will save lives,^6^, ^7^, ^8^ several high‐profile publications have asserted that there is a “U”‐ or “J”‐shaped curve, in which increased mortality is associated with both the highest and the lowest levels of sodium.^4^, ^9^, ^10^ Careful analysis and interpretation of the totality of available data, including recognition of potential conflicts of interest, is needed to place appropriate focus on the most valid evidence.

High sodium intake over a lifetime is related to the age‐related rise in blood pressure which is observed in most populations. However, in populations with very low salt intake, blood pressure does not increase with age.^3^ Sodium intake recommendations have traditionally relied on blood pressure as a surrogate end point for cardiovascular disease.^6^ The highest quality trials on the impact of sodium reduction on blood pressure, such as the DASH‐Sodium trial, randomize participants to controlled diets with different levels of sodium and find a direct progressive relationship between sodium intake and blood pressure.^11^ These conclusions were recently affirmed in the US National Academies of Sciences, Engineering, and Medicine's *Dietary Reference Intakes for Sodium and Potassium*, a rigorous assessment designed to identify the recommended amount of sodium to reduce chronic disease risk,^6^ and by a recent review by the European Food Safety Authority.^8^ The National Academies found (1) a high strength of evidence, based on more than 35 randomized controlled trials, that reducing sodium intake reduces blood pressure, and (2) a moderate strength of evidence, based on five trials of at least one year in length, that reducing sodium reduces hypertension incidence, cardiovascular events, and all‐cause mortality.^6^

### 2.1 Sodium intake measurement error

It can be extremely challenging to conduct high‐quality trials to assess the relationship between sodium intake and cardiovascular outcomes. Such trials would require a large number of participants as well as a sustained reduction in sodium intake for several years; without changes in the food supply, the necessary behavioral change has proven difficult to maintain for longer than 6 months.^12^ This gap has led to a proliferation of observational studies; these studies unfortunately are prone to systematic error in the measurement of sodium intake. The most accurate measurement is at least three non‐consecutive 24‐hour urine samples.^13^ In contrast, most observational studies that have reported a U‐ or J‐shaped relationship with mortality have not used even a single 24‐hour sample.^9^, ^10^, ^14^ Alternative means of estimating sodium, such as dietary questionnaires and spot urine samples, are highly inaccurate.^13^, ^15^

Studies that estimate usual sodium intake from spot urine samples are particularly problematic. Compared with 24‐hour urine collection, estimates from spot urine samples overestimate intake among people with low sodium intake and underestimate intake among people with high sodium intake, sometimes substantially.^13^ Together, this can create an artifactual appearance of increased mortality at apparently low levels of sodium intake. Furthermore, these studies use formulas to estimate usual daily intake^6^, ^9^, ^13^ which rely on age, sex, and urine creatinine, all of which are independently associated with cardiovascular disease and which can therefore introduce an apparent but non‐causal J‐ or U‐shaped relationship.^13^, ^16^

**FIGURE 1:**
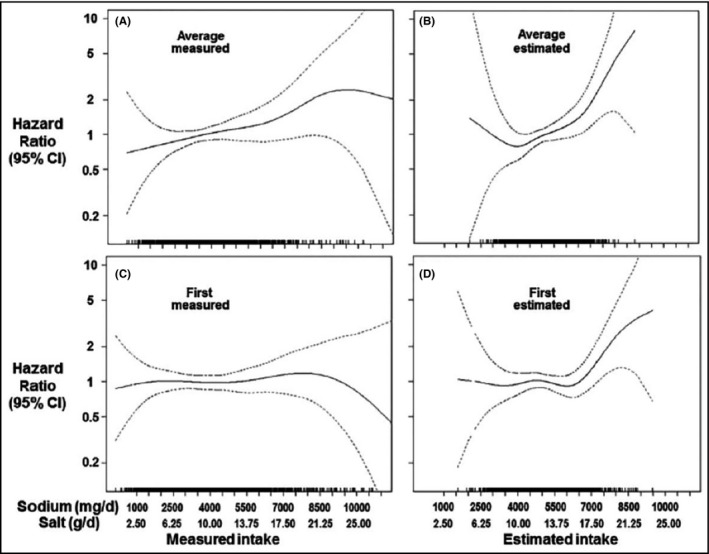
Impact of sodium measurement on perceived relationship between usual sodium intake and CVD. Source: He FJ, Campbell NRC, Ma Y, MacGregor GA, Cogswell ME, Cook NR. Errors in estimating usual sodium intake by the Kawasaki formula alter its relationship with mortality: implications for public health. *Int J Epidemiol*. 2018;47(6):1784‐1795.^17^ The four graphs represent different spline plots on the association between sodium intake and all‐cause mortality in the Trials of Hypertension Prevention (TOHP) study after 20 years of follow‐up. In each graph, the measurement of sodium intake varies, resulting in a change in the relationship between sodium intake and all‐cause mortality. Sodium intake is measured as (A) the gold standard of the average sodium from 3 to 7 collections of 24‐h urine, (B) the average of 3‐7 *estimated* 24‐h urine excretion based on applying the Kawasaki formula to the sodium concentration, (C) the sodium measured in the first 24‐h urine collection, and (D) the sodium *estimated* from a applying the Kawasaki formula to the sodium concentration in the first 24‐h urine collection. Reprinted with permission

Although the above considerations are theoretical, He et al's recent study provides powerful empiric evidence that the J shape is an artifact and does not represent an actual causal relationship (Figure 1).^17^ Using long‐term data from the Trials of Hypertension Prevention follow‐up study, a trial in which people were randomized to reduce sodium intake for 18‐48 months, they found that the linear relationship between measured sodium intake (based on three to seven 24‐hour urine samples) and mortality changed to an artifactual J‐shaped relationship when estimation equations commonly applied to spot urine samples were used. As the Figure shows, the actual linear relationship (solid line, Panel A) becomes an artifactual J shape (solid line, Panel B) when the equation is used, and the actual linear relationship documented with multiple 24‐hour specimens is attenuated when a single 24‐hour specimen is used (solid line, Panels C and D).

Even studies that rely on a single 24‐hour urine collection can lead to incorrect inferences. Olde Engebrink et al found significant misclassification when a single, baseline 24‐hour urine collection was used compared to a mean of multiple measurements across years. With one measurement, the relationship between 24‐hour urine sodium and cardiovascular events and mortality appeared to be J‐shaped; multiple measurements documented the actual progressive direct relationship.^18^ Thus, both studies documented rigorously that the J shape observed in some reports is an artifact of inaccurate estimation of sodium intake.

### 2.2 Reverse causality

Many observational studies that reported a J‐ or U‐shaped relationship also included people with illnesses such as cardiovascular disease, kidney disease, and diabetes. Because illness can lead participants to alter their diet, sicker participants often have lower sodium intake and are also more likely to die during the follow‐up period (reverse causality).^14^ A similar J‐shaped relationship due to reverse causality is the apparent but non‐causal association between low blood pressure and increased mortality. This is due to a decline in blood pressure among patients whose health is failing rather than a causal relationship.^19^

## 3 INTERVENTIONS

The science is clear: Reducing sodium consumption reduces blood pressure and the risk of cardiovascular events. The more difficult challenge is implementing population‐wide sodium reduction interventions that can save millions of lives.^2^ As part of the Resolve to Save Lives Cardiovascular Health Initiative, we aim to build on global best practices to implement and scale up promising programs to reduce population sodium intake in low‐ and middle‐income countries. Finland, Japan, and the United Kingdom have reduced population sodium intake and achieved associated reductions in both blood pressure and cardiovascular disease.^3^, ^20^ South Korea recently reported reductions in sodium intake and blood pressure.^21^ Effective sodium reduction initiatives must be both scalable and aligned with the major sources of sodium in each country (ie, packaged food, food prepared and cooked in the home, food prepared outside of the home). Multi‐component programs with a structural or policy component (eg, regulation, taxation) have been most effective.^20^

### 3.1 Packaged food

Tested, scalable approaches exist for reducing sodium in packaged food, which is the major source of sodium in most high‐income countries. The most well‐known establishes food‐category‐specific targets for sodium, as pioneered by the United Kingdom. Their quasi‐mandatory program led to a 15% decrease in salt intake and a drop in hypertension and CVD between 2003 and 2011.^22^ More recently, mandatory limits for sodium in specific food categories have been implemented in Argentina and South Africa, though the impact on sodium intake has not yet been documented.

Emerging evidence suggests that mandatory front‐of‐pack warnings, as implemented in Chile for foods high in sugar, salt, saturated fat, or calories, may be a highly effective strategy. Chile's 24% decrease in purchases of high‐sugar beverages is suggestive^23^; evaluations of the impact on sodium are ongoing. Such warnings empower consumers to select healthier options and encourage companies to reformulate to avoid the warning requirement threshold. Finally, some countries have begun to consider taxes on high‐salt foods to reduce sodium intake, following the model applied to sugary beverage taxes. Strategies that reduce consumption of unhealthy food often elicit vigorous opposition from the processed food industry; however, this should not dissuade governments from adopting them.

### 3.2 Away‐from‐home foods

Away‐from‐home foods are an increasingly important source of sodium intake globally. Many jurisdictions have had success increasing the healthfulness of food purchased and served by governments in schools, hospitals, and workplaces through nutrition standards, including sodium content limits.^24^ These policies have potentially large reach and, because they do not directly regulate industry, may meet less opposition.

In contrast, there are few tested, scalable interventions for settings such as restaurants or street food vending. Approaches similar to those for packaged food—with targets for steady reduction and warning labels on high‐sodium food—can be applied to chain restaurants but have not been evaluated. Innovation is needed to identify interventions that can reduce sodium substantially in these settings.

### 3.3 In‐home food preparation

Approaches are also needed that reduce salt added to food during cooking or at the table, the primary source of sodium in many low‐ and middle‐income countries. The most common way to address salt used in the home is through consumer education. Unfortunately, rigorous evaluations of educational interventions are rarely performed; of the evaluations which have been done, null findings or negligible change is common.^20^, ^25^ There is an opportunity to learn lessons from global tobacco control programs, in which mass media campaigns had an impact on behavior.^26^ Social media approaches also have the potential to reach many people, but the impact and duration of behavior change has not yet been properly evaluated.

Promoting and/or subsidizing potassium‐enriched salts (salt containing 10%‐50% potassium chloride) to encourage large‐scale adoption for home use is a promising approach. Strong evidence from randomized trials shows that these salts reduce blood pressure,^27^ and an ongoing trial, the China Salt Substitute and Stroke Study, is expected to provide results by 2022 (the 5‐year study endpoint) on the direct relationship of salt substitutes with cardiovascular disease.^28^ These salts were introduced decades ago but have not been promoted for widespread use. Because of potential hyperkalemia, people with renal disease and those on treatment for hypertension with renin‐angiotensin system inhibitors including angiotensin‐converting enzyme inhibitors and angiotensin‐receptor blockers should be advised to consult their doctor before using potassium‐enriched salt. However, given that the amount of potassium consumed in low‐sodium salts would rarely exceed the equivalent of 1‐2 bananas a day, concern about potential hyperkalemia need not deter efforts to promote consumption of these products. A recent modeling study estimated that in China, the benefits on cardiovascular disease of substituting table salt with a potassium‐enriched salt greatly exceeded the potential harm of hyperkalemia.^29^

## 4 CONCLUSION

Progress in sodium reduction has been limited to high‐income countries that have implemented multi‐component interventions and where packaged food is the major source of sodium. For many low‐ and middle‐income countries where home‐cooked food is the leading source of sodium, multi‐component interventions will still be critical. To address home‐cooked foods, mass media campaigns, behavioral change interventions, and the use of potassium‐enriched salt are under active exploration. Further work is needed to determine effective interventions to reduce the sodium content of restaurant and street foods.

The evidence strongly indicates that sodium reduction can decrease cardiovascular disease. Now the focus must be on implementing the most suitable strategy, tailored to the specific circumstances of each country, that would best reduce salt intake and prevent millions of premature deaths.

## CONFLICT OF INTEREST

Nothing to disclose.

## AUTHOR CONTRIBUTIONS

Each of the listed authors (LKC, TRF, and LJA) meet the criteria for “Authorship” in accordance with the ICMJE recommendations as outlined below: Substantial contributions to the conception or design of the work; drafting the work or revising it critically for important intellectual content; final approval of the version to be published; and agreement to be accountable for all aspects of the work in ensuring that questions related to the accuracy or integrity of any part of the work are appropriately investigated and resolved.
